# Electric field created p–n junction in composite films made from carbon nanotubes, iron (III) sulfate and polyvinyl alcohol

**DOI:** 10.1038/s41598-022-15294-4

**Published:** 2022-07-01

**Authors:** Hsin-Jung Tsai, Ching-You Ke, Yung-Kai Yang, Wen-Kuang Hsu

**Affiliations:** Department of Materials Science and Engineering & High Entropy Alloy Center, National Tsing-Hua University, Hsinchu City, 300044 Taiwan

**Keywords:** Materials science, Nanoscience and technology

## Abstract

Suspension made of Fe_2_(SO_4_)_3(*aq*)_, polyvinyl alcohol and carbon nanotubes is placed in electric field to separate charges. Charges remain separated as suspension solidifies, forming composite films with cations and anions enriched at opposite sides. Polarized films behave as junction diodes with forward current and threshold voltage found to be 10^–4^–10^–5^ A and 2.4–2.6 V at ± 5 V. Rectification is preserved in strained composite films.

## Introduction

Diodes consist of two different semiconductor materials and produce one-direction current (*e.g*. p–n junctions). Attributed to equalization of Fermi level, the band bends and a built-in-potential (ϕ) forms at interface where density of free carriers is as low as an insulator^1^. This insulating region, also known as depletion layer (DL), becomes widened in reverse bias voltage. In this case, the ϕ rises and current can barely pass before breakdown. Upon forward bias, interfacial barrier is reduced and current flows at low voltage. However, production of junction diodes is lengthy and involves use of organic solvents and toxic elements^2^. Conductive polymers (CPs) represent a new type of electronic materials and can be classified into intrinsic (type-I) and extrinsic (type-II); the former is a type of thermoset and transports carriers through conjugated networks (*e.g.* polyacetylene)^3^. The latter requires conductive fillers that form networked paths at percolation threshold (η). Accordingly, the type-II can use both thermoplastic and thermoset polymers as matrix and has become the subject of great interest in materials science community^4,5^. This work demonstrates for the first time the generation of type-II based p–n junctions where DL is created by applying elecric field (***E***) to suspension made of electrolytes, polymer and carbon nanotubes (CNTs). Composite based thin film diodes are highly flexible and can be used as sensing devices, e.g. blood pressure detection and humidity controlled sensor^1^.

## Experimental

### Suspension of Fe_2_(SO_4_)_3(*aq*)_/polyvinyl alcohol/multi-walled carbon nanotubes

**Figure 1:**
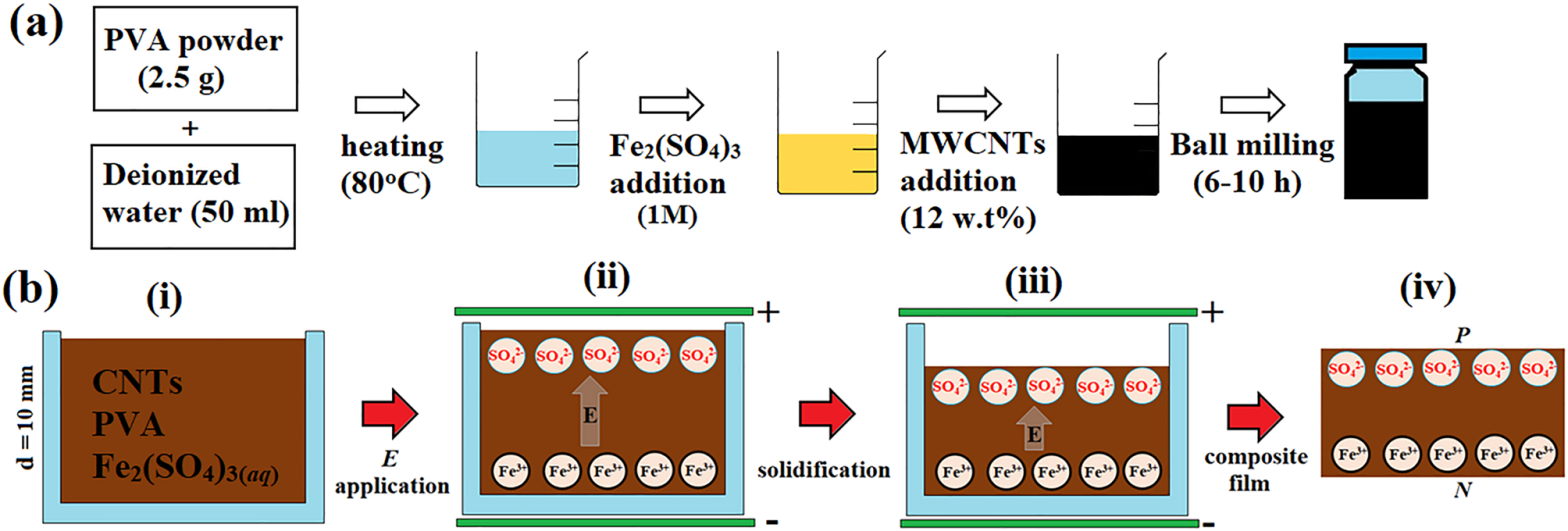
Preparation of PVA/Fe_2_(SO_4_)_3*(aq)*_/CNTs (**a**) and ***E*** treatment of suspension until solidification (**b**).

Polyvinyl alcohol (PVA) does not thermally decompose before 120 °C and, attrubuted to hydrophilic and hydrophobic moieties from alkyl and hydroxyl, exhibits affinity to both CNTs and electrolytes. CNTs, on the other hand, are one-dimension conductor made of rounded graphite sheets. Accordingly, tube strength and conductivity display anisotropy and are mainly contributed by C–C bonds along tube axis^6^. Owing to their high aspect ratio and formation of percolation paths at low mass filling fraction (*f*_*CNT*_), CNTs are now widely used as conductive fillers to make type-II CPs^7^. Similar to PVA, CNTs also exhibit an amphiphilicity and can strongly interact with ions in aqueous solution^8^. In this work, multi-walled CNTs (MWCNTs) are introduced into Fe_2_(SO_4_)_3_-PVA composites to improve electrical conductivity and ion adsorption. PVA powders (2.5 g, MW = 120,000, CCP, LTD) and Fe_2_(SO_4_)_3(*aq*)_ (SHOWA) are dissolved in deionized water (50 ml) at 80 °C. MWCNTs made by catalytic pyrolysis of hydrocarbons (> 95% purity, tube diameter and length = 10–50 nm and 15–30 μm, Integrated Bio, LTD) are added into polymer-electrolyte solution, followed by ball milling for 10 h at room temperature (Fig. 1a). Previous workers claim that ball milling may damage tube structure and broadens tube length distribution accordingly^9^. Here SEM and raman reveal structural change to be minor; the I_D_/I_G_ being 1.38 before and 1.41 after treatments (supplementary information 1a-d). Suspensions made of different electrolyte concentrations (***el***-***Conc***) and *f*_*CNT*_ are prepared where ***el***-***Conc*** = 0.1, 0.3, 0.5 and 1 M and *f*_*CNT*_ = 2.5, 5, 7.5, 10, 12.5 and 13 wt%.

### DL creation by *E* application to suspensions

The DL is created according to following procedures. First, suspension is transferred to a petri dish placed between cupper plate electrodes at room temperature (i-ii, Fig. 1b). Second, electrodes are electrified so ***E*** forms and Fe^3+^ and SO_4_^2−^ are separated to opposite sides of suspension (ii, Fig. 1b). Since spacing between plates is fixed (d = 1 cm) the intensity of ***E*** is simply expressed as V/cm according to uniform field theory ***E*** = V/d. Third, ***E*** is applied until suspension solidifies and becomes composite films (iii-iv, Fig. 1b). Fourth, composites films made with and without ***E*** treatments are compared. Note all resultant composite films have been kept in oven (60 °C, 24 h) and weighed before and after to calculate the residual water content (~ 0.1 wt%).

### Structural and electrical characterizations of composite films

As made composite films are inspected by optical and scan electron microscope (OM equipped with precision measurement s ystem & SEM, Hitachi-SU8010) which give information regarding to film thickness, surface texture and CNT distribution. The current–voltage (I–V) profiles, electrical conductivity (σ) and resistivity (ρ), normalized ρ at 25 °C (ρ/ρ_25°C_), forward current (I_F_), threshold voltage (V_th_), revere and forward device resistance (***R***_***R***_ and ***R***_***F***_) are measured using a semiconductor device analyzer equipped with mobile-probe system (± 5 V, Agilent B1500A). It is worth mentioning for conventional devices that DL suffers greatly from heating according to Joule’s laws expressed as P = VI and I = Q/t where P, V. I, Q and t denote power, voltage, current, thermal energy and time. Accordingly, the I_F_ is defined as the maximum forward current before onset of thermal damage^1^. The ***R***_***R***_/***R***_***F***_ ratio, on the other hand, is a key parameter often used to characterize electrical rectification, namely, ***R***_***R***_/***R***_***F***_ ≈ 1 indicates ohmic conductors (i.e. symmetric I-V profile) and ***R***_***R***_/***R***_***F***_ > 1 for polarized conductors^1^. All electrical measurements are carried out at a low relative humidity (RH = 25%) to evade H_2_O doping since both PVA and CNTs are electronically sensitive to moisture^10^.

### Elemental analyses

X-ray photoelectron emission spectroscopy (XPS, ULVAC-PHI PHI 5000 Versaprobe II) is employed to probe distribution and content of S and Fe at film surfaces where ***P*** and ***N*** denote positively and negatively charged sides (iv, Fig. 1b). XPS peak intensity (**I**_**t**_) is calculated according to full width at half maximum (FWHM) and signal intensity (C_*x*_) of S2p and Fe2p spectra expressed as C_*x*_ = n_*x*_/∑n_*i*_ = (I_*x*_/X_*x*_)/∑(I_*i*_/X_*i*_) where X_*x*_ is corrected relative sensitivity factor and I_*x*_ is signal intensity^11^. The ***P***/***N*** ratio which is based on C_*x*_ indicate the level of ***E*** induced film polarization, *i.e. ****P***/***N*** ≅ 1 for non-polar and, ***P***/***N*** > 1 or < 1 for polar.

### Work function measurements

The work function (Φ) of composite films is calculated according to Φ = *hv*—E_o_—E_F_ where excitation energy (*hv*), cut-off energy (E_o_) and Fermi energy (E_F_) are determined by tangential equation and ultraviolet photoelectron spectroscopy (UPS) at *hv* = 21.22 eV^12^. Supplementary information 2a-c shows UPS spectra where E_o_, E_F_ and Φ are found to be 13.2 and -3.2 and 4.91 eV. For the sake of clarity, the Φ and UPS are only calculated for samples made of ***el***-***Conc*** = 0.1 and 0.5 M at ***E*** = 0 and 600 V/cm (supplementary information 2d-h). The obtained Φ is then used as reference to evade formation of Schottky potential at lead-sample interface, namely, the Φ of metal leads must be lowered and greater respectively than ***P*** and ***N*** of samples^13^. Study here selects Ag (Φ = 4.26 eV) and Pt (Φ = 5.65 eV) paste as metal leads in connection with ***P*** and ***N***.

### Molecular dynamic calculations

Molecular dynamic (MD) calculation is performed to characterize ***E*** induced charge separation in suspension and, single-walled CNTs (SWCNTs) are used as modelling matrix for the following reasons. First, less carbon atoms are invovled so calcualtion time is shortened. Second, SWCNTs resemble MWCNTs in terms of adsorption, transport, chemical reactivity and optical properties^14^. Two (5,0) SWCNTs (2 × 240 = 480 atoms), 3 PVA molecules [(CH_2_–CHOH)_n_, n = 20], 50 H_2_O, 40 Fe^3+^ and 60 SO_4_^2−^ are built in a 3 × 3 nm cell with ionic ratio of 40:60 according to net equation Fe_2_(SO_4_)_3_ → 2Fe^3+^  + 3SO_4_^2−^. Calculations are carried out on three different structures defined as complex-I (50 H_2_O + 40 Fe^3+^ + 60 SO_4_^2−^), complex-II (50 H_2_O + 40 Fe^3+^  + 60 SO_4_^2−^ + 3 PVA) and complex-III (50 H_2_O + 40 Fe^3+^ + 60 SO_4_^2−^ + 3 PVA + 2 CNTs). Each is geometrically optimized using NVT ensemble for 500 ps at 300 K and ***E*** = 600 V/m. The COMPASS force field is used for MD simulations and, the Non-Bond Task apply settings are based on Waals & Coulomb force. The cutoff distance, spline and buffer width are set at 12 Å, 1 Å and 0.5 Å.

## Results and discussion

**Figure 2:**
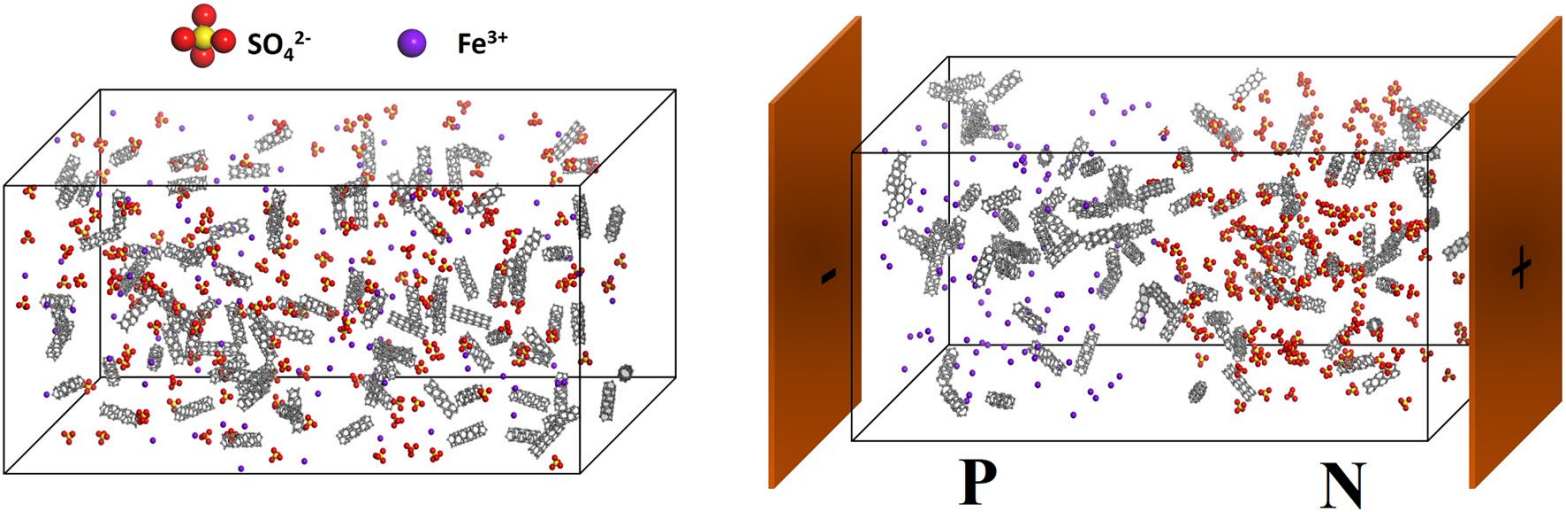
Scheme of DL creation by ***E*** application. Suspension consisting of PVA, Fe_2_(SO_4_)_3*(aq)*_ and CNTs at ***E*** = 0 (left) and ***E*** > 0 (right).

**Figure 3:**
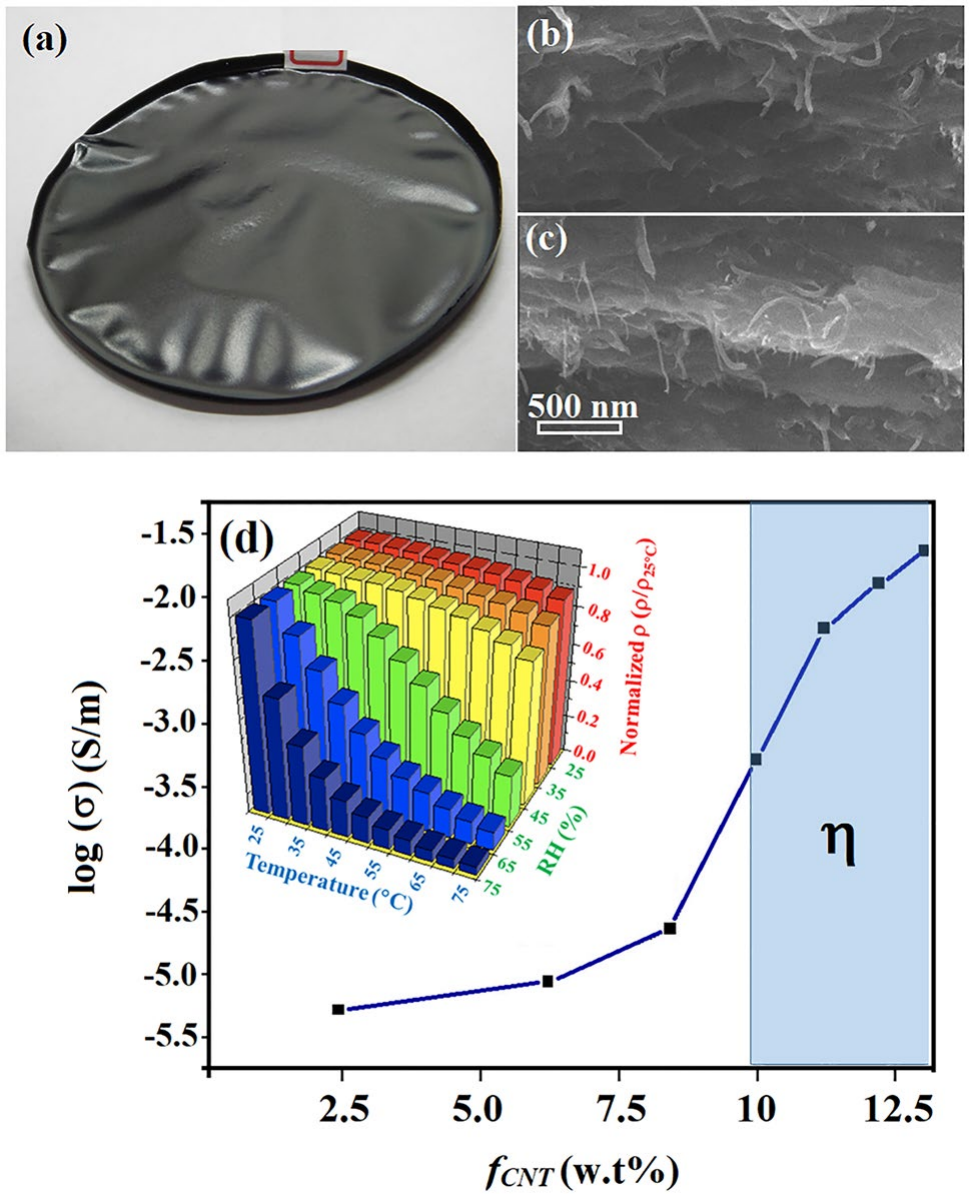
Optical image of as-made (**a**) and SEM images obtained from cross-section (**b**–**c**). The logσ-*f*_*CNT*_ profile (**d**) and plots of normalized ρ v.s. T and RH (insert). Blue region denotes *f*_*CNT*_ = η.

Conventional devices are made by chemical doping to create DL where the number of uncompensated carriers is dependent of doping level. Even doping of p and n impurities gives step junctions with ϕ = ***E***_max_*w*/2 and N_A_ = N_D_ where ***E***_max_, *w*, N_A_ and N_D_ denote the maximum ***E*** at interface, width of DL, the number of acceptor and donor carriers at DL. Abrupt junction however forms if doping is uneven. In this case, N_A_ ≠ N_D_ and *w* = [2ε_s_/*e*·(N_A_ + N_D_)/(N_A_·N_D_)·ϕ]^1/2^ where ε_s_ and *e* are permittivity and electron charge. Instead of chemical doping, the DL here is created by separating Fe^3+^ and SO_4_^2−^ in composite films (Fig. 2) so formation of abrupt junction is unlikely and all electrical parameters are studied on the basis of step junction. Figure 3a displays a typical example of composite films with thickness measured to be 1 mm. Clearly, CNTs are well-dispersed and mostly embedded according to SEM images obtained from different regions of cross-section (Fig. 3b–c). The log(σ)-*f*_*CNT*_ plot indicates *f*_*CNT*_ = η = 10–12 wt% at which the σ is 2–3 orders of magnitude greater than value obtained at *f*_*CNT*_ = 2.5 wt% (Fig. 3d). The small fluctuation of ρ/ρ_25°C_ with rising temperature at RH = 25%, however, excludes H_2_O doping (insert, Fig. 3d)^10^.

**Figure 4:**
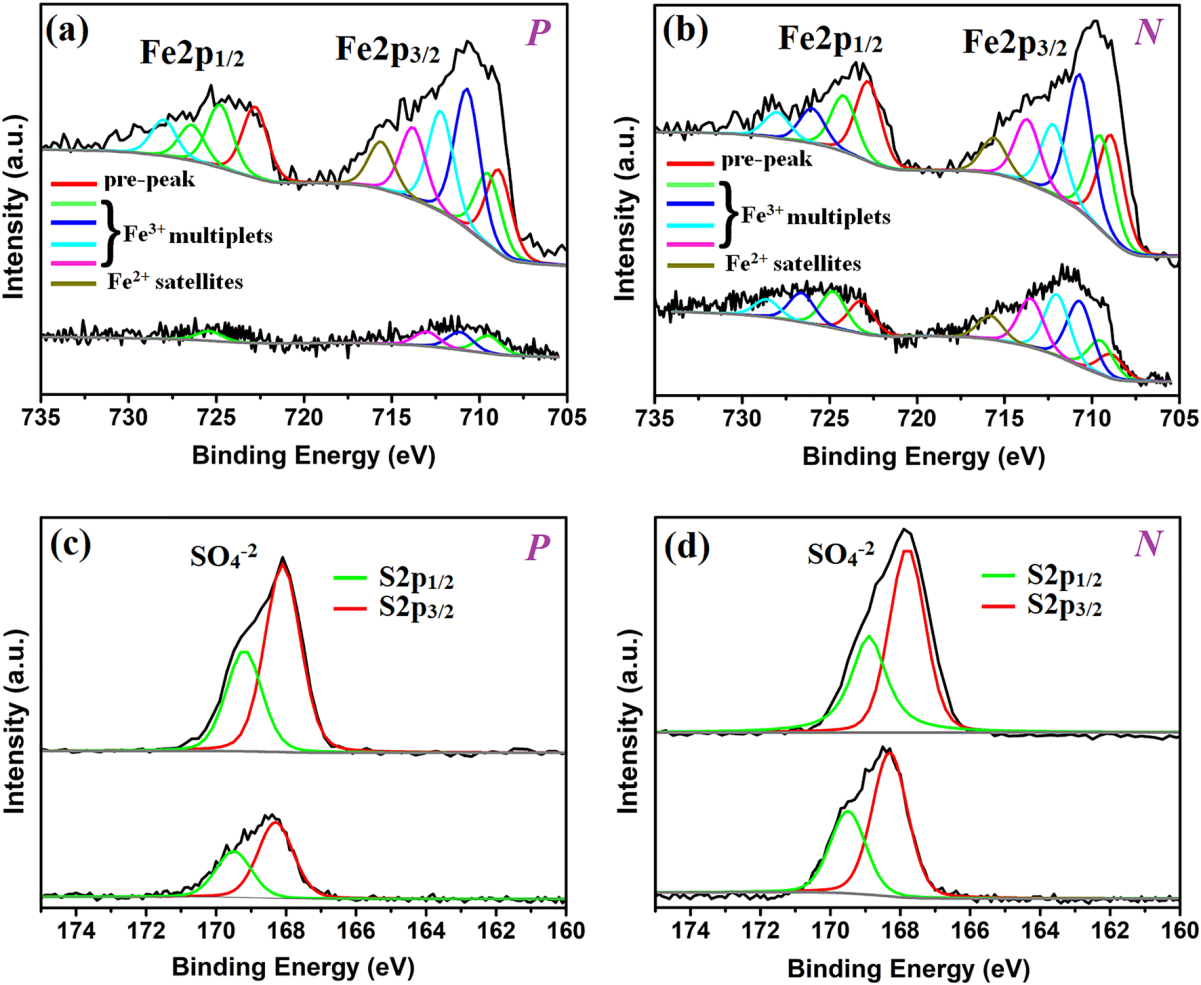
XPS spectra of Fe2p at ***P*** (**a**) ***N*** (**b**) and S2*p* at ***P*** (**c**) and ***N*** (**d**). Top and lower profiles denote samples with and without CNTs.

Figure 4a-d shows XPS profiles where ***P***-Fe2p, ***P***-S2p, ***N***-Fe2p and ***N***-S2*p* denote Fe2*p* and S2*p* spectra recorded at ***P*** and ***N*** of composite films made from ***el***-***Conc*** = 1 M and ***E*** = 30 V/cm. The Fe2*p*_1/2_ and Fe2*p*_3/2_ are present in ***P***-Fe2*p*; the former consists of Fe^3+^multiplets (singlet, 722–730 eV) and Fe^2+^ satellite for the latter (triplet, 707–717.5 eV, Fig. 4a)^15^. We find that CNT addition truly promotes ion absorption thus giving a greater **I**_**t**_ compared with CNTs-free samples (top and lower, Fig. 4a). Detection of Fe^2+^, on the other hand, indicates Fe^3+^ → Fe^2+^ reduction through CNTs acting as reductant^16^. Similar profiles also appear at ***N*** with (i) **I**_**t(*P*)**_ < **I**_**t(*N*)**_ for CNTs-free (lower, Fig. 4b) and (ii) **I**_**t(*P*)**_ ≈ **I**_**t(*N*)**_ for CNTs loaded films where **I**_**t(*P*)**_ and **I**_**t(*N*)**_ denote XPS peak intensity at ***P*** and ***N*** (top, Fig. 4b). The (i) implies that cations move with ***E*** and become enriched at ***N***. The (ii) is due to ion-tube physisorption thus reducing mobility of ions toward electrodes^8^. In S2*p* spectra, sulfates (SO_4_^2−^) appear in the form of doublet [167.9 eV (S2*p*_3/2_) and 169 eV (S2*p*_1/2_)], attributable to spin–orbit interaction (Fig. 4c–d)^15^. Again, **I**_**t(*P*)**_ ≈ **I**_**t(*N*)**_ and **I**_**t(*P*)**_ < **I**_**t(*N*)**_ are present respectively in films with and without CNT addition (top and lower, Fig. 4c and d). Table 1 lists ***P***/***N*** ratio and content of Fe and S for composites made at different ***E***. First, samples made at ***E*** = 0 exhibit fluctuations at 0.97–1.15 for S and 0.55–1.09 for Fe, attributable to random diffusions of ions in solution^17^. The ***P***/***N*** then stabilizes at 1 for samples made at ***E*** = 30 V/m. Second, ***P***/***N*** = 1.14 (S) and 0.75 (Fe) appears at samples produced at ***E*** = 600 V/m, verifying charge separation to opposite sides of composite films (*i.e. ****P***/***N*** ∝ ***E*** for S and ***P***/***N*** ∝ ***E***^−1^ for Fe). It is worth mentioning in wafer technology that junction is created by doping thus producing a concentration gradient of dopants along DL. Here micro-analyses along film thickness do not verify a concentration gradient of cations (supplementary information 3), again supporting DL creation through film polarization (*i.e. ****P***/***N*** > 1 or ***P***/***N*** < 1 at surfaces).

**Table 1:** *P*/*N* ratio and content of Fe and S at different *E*.

E (V/cm)	location	S (%)	*P/N*	Fe (%)	*P/N*
0	*P*	76.89	0.97–1.15	23.11	0.55–1.09
0	*N*	78.87		21.13	
30	*P*	68.17	1.00	31.83	1.00
30	*N*	68.14		31.86	
600	*P*	73.58	1.14	26.42	0.75
600	*N*	64.72		35.28	

**Figure 5:**
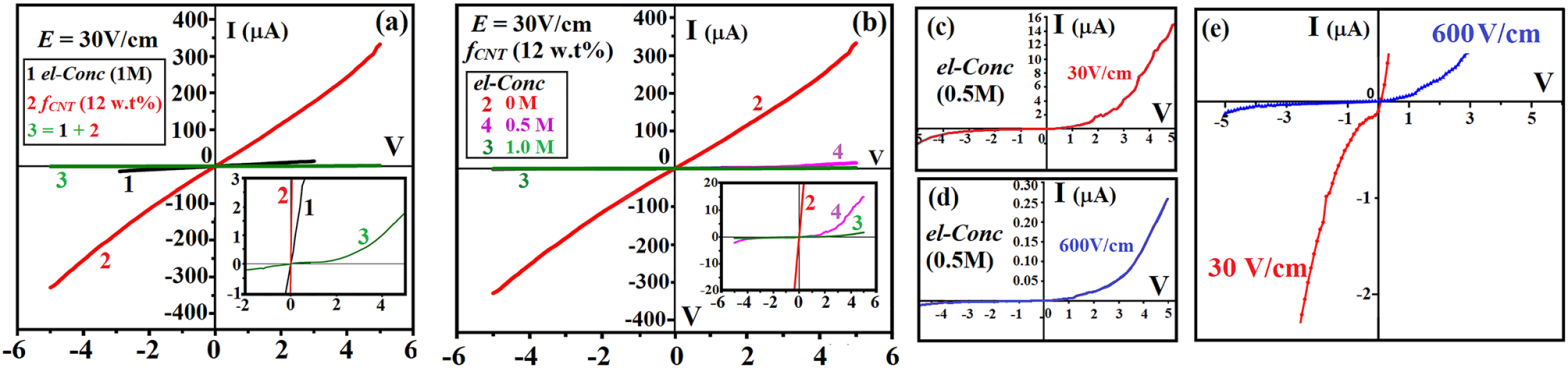
The I–V profiles of samples **1**, **2** and **3** at ± 6 V and highlighted profile at ± 4 V (**a**). The I–V profiles of samples **2**, **3** and **4** at ± 6 V and highlighted profile at ± 4 V (**b**). Highlighted I–V profiles of sample **4** made at ***E*** = 30 V/cm (**c**) and 600 V/cm (**d**). Highlighted I–V profiles of (**c**) and (**d**) at ± 5 V and I =  ± 2 μA (**e**).

Figure 5a shows I–V profiles of composite films made from PVA loaded respectively with electrolyte (***el****-****Conc*** = 1 M, sample **1**) and CNTs (*f*_*CNT*_ = 12wt%, sample **2**) at ***E*** = 30 V/cm. Addition of both electrolyte and CNTs into PVA yields sample **3** (i.e. samples **1** + **2**). Clearly, samples **1** and **2** exhibit a linear I–V character; the latter due to networking of CNTs at η exhibits a lower ***R*** (insert, Fig. 5a)^18^. Sample **3**, on the other hand, resembles a diode (insert, Fig. 5a). Diode character is enhanced as ***el***-***Conc*** of sample **3** is reduced to 0.5 M (defined as sample **4**, insert, Fig. 5b). Data above indicates following, first, CNTs provide samples with σ (i.e. sample **2**), second, diode character is only present in samples made from electrolyte, CNTs and ***E*** treatment (i.e. samples **3** and **4**). Third, diode profiles vanish when CNTs or electrolytes are removed from sample **3** (*i.e.* samples **1** and **2**). Fourth, the optimal ***el***-***Conc*** for diode production is 0.5 M (i.e. sample **4**). I-V measurements are then carried out on samples **5** and **6** to probe optimal ***E*** for diode production where **5** and **6** denote composite films made by addition of ***el***-***Conc*** = 0.1 and 0.3 M respectively into sample **2** (supplementary information 4a–b). Clearly, diode character reoccurs in samples **5** and **6** with I_F_ measured to be 310 μA and 75 μA at 5 V (***E*** = 600 V/cm). Highlighted I–V profiles of sample **6** further reveal an enhanced diode character where V_th_ lies 2.4–2.6 V at ***E*** = 600 V/cm (supplementary information 5a-c). Note that DL is a polarized region with field vector against external ***E*** the I_F_ is therefore expected to be lower than a conductor, accounting for I_F(***E*** = 0V)_ = 120 μA > I_F(***E*** = 30V)_ = 85 μA > I_F(***E*** = 600V)_ = 75 μA at 5 V (supplementary information 5a–c). Additional evidence in support of film polarity comes from the Φ measurements (Table 2). First, Φ_***P*(600 V)**_ > Φ_***N*(600V)**_ is present where Φ_***P***_ and Φ_***N***_ denote Φ obtained at ***P*** and ***N***. Second, Φ rises as ***el***-***Conc*** increases from 0.1 to 0.5 M, indicative of enriched ions at surfaces. However, the Φ varies significantly from region to region as ***el***-***Conc*** reaches 1 M (not shown), attributed to excessive ions that diffuse randomly thus compromising DL formation^17^. Supplementary information 6 shows stability of diode characteristic over 12 months kept in the oven.

**Table 2:** Variation of Φ with ***E*** at different ***el***-***Conc***.

*el-Conc*	Φ		
	0 V/cm	30 V/cm (*P*)	600 V/cm (N)
0.1 M	4.91	5.12	4.52
0.5 M	5.22	5.52	4.92

Diode performance is often justified according to quality factor and ***R***_***R***_/***R***_***F***_ ratio^19^; the former is used to characterize doped devices and is inapplicable to CPs. The latter concerns unidirectional conduction so V_th_ must low and high I_F_ is required, for example, V_th_ = 0.7 V and I_F_ = 10^–3^–10^–5^ A for Si-based diodes^20^. Experiments indicate that diode character becomes apparent at ***R***_***R***_/***R***_***F***_ > 3 and, the V_th_ and I_F_ lie 2.4–2.6 V and 10^–4^–10^–5^ A for samples made at ***E*** = 30 and 600 V/cm (Table 3 and Fig. 5c–e). The ***R***_***R***_/***R***_***F***_ measurements also confirm optimal conditions for diode formation to be *f*_*CNT*_ = 12 wt%, ***el***-***Conc*** = 0.5 M and ***E*** = 600 V/cm; the greatest value being ***R***_***R***_/***R***_***F***_ = 20.663 (Table 3). In CMOS technology, the Si substrates are intentionally stressed to break crystallographic symmetry thus altering band structure^21^. However, strains are confined at interfaces and can barely propagate to detriment conductive channels underneath. We find that film area and thickness increase and decrease respectively by 200% and 80% after hot-pressing (60 °C, 50 kg/cm^2^ and 30 min) and the ***R***_***R***_/***R***_***F***_ changes to 3.176 (***E*** = 0), 2.358 (***E*** = 30 V/m) and 4.03 (***E*** = 600 V/m), indicating redistribution of ions and CNTs (Table 3 and supplementary information 7a–b).

**Table 3:** Variation of ***R***_***R***_/***R***_***F***_ with ***E*** at different ***el***-***Conc***.

*el-Conc*	*R*_*R*_*/R*_*F*_		
	0 V/cm	30 V/cm	600 V/cm
0.1 M	1.056	2.024	2.804
0.3 M	1.344	1.134	3.979
0.5 M	1.287	7.054	20.663
1 M	1.365	3.744	7.068
Hot pressed (0.3 M)	3.176	2.358	4.03

**Figure 6:**
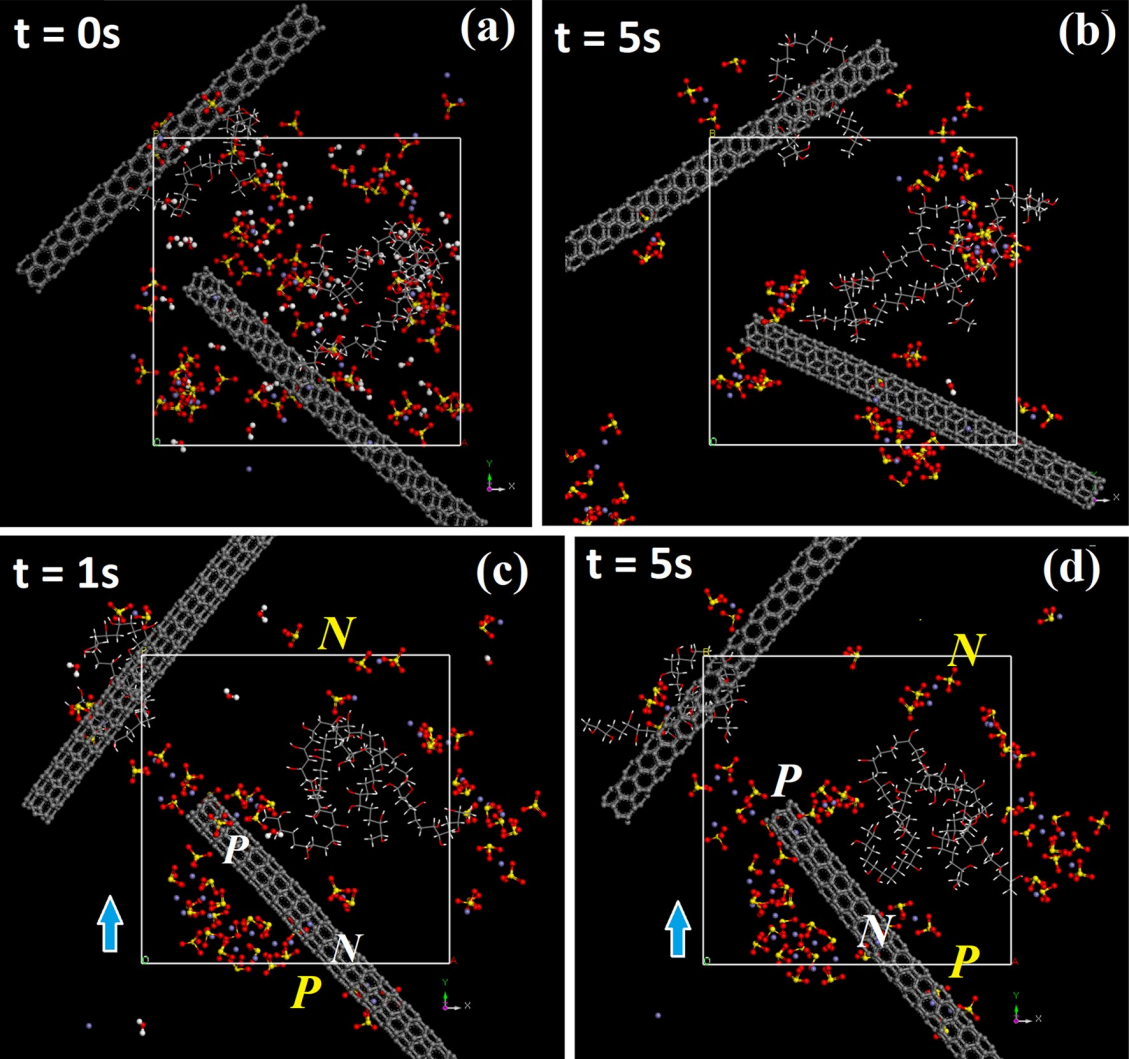
Snapshots extracted from geometric optimization of complex-III at ***E*** = 0 and t = 0 s (**a**) and 5 s (**b**) and at ***E*** = 600 V/cm and t = 1 s (**c**) and 5 s (**d**). Polarized tubes display ***E*** in opposite direction to external field (***P*** and ***N*** in white).

**Figure 7:**
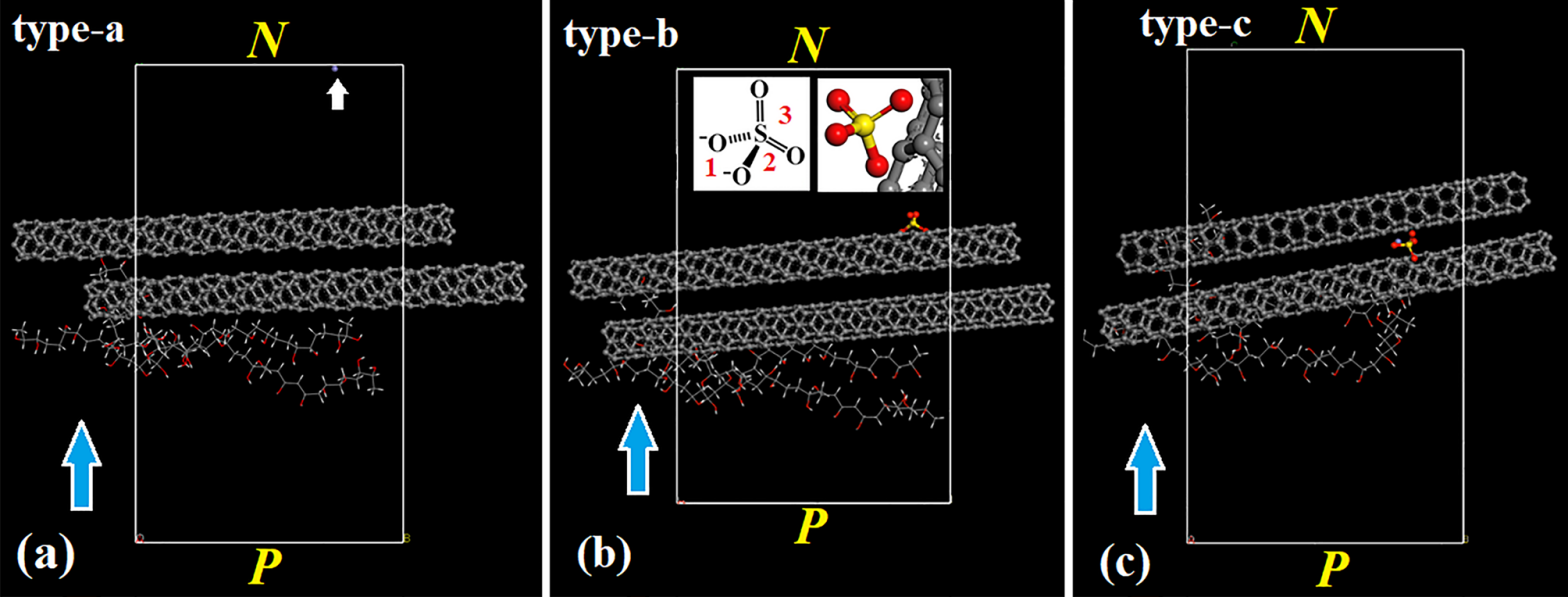
Snapshots extracted from geometric optimization of **type**-**a** at ***E*** = 600 V/cm and t = 1 s (**a**), **type**-**b** at ***E*** = 600 V/cm and t = 1 s (**b**) and **type**-**c** at ***E*** = 600 V/cm and t = 1 s (**c**).

Question however remains as to how charges separate in the presence of ***E***, CNTs and PVA. Supplementary information 8 shows consecutive snapshots extracted from MD simulations of complex-I which contains three types of intermolecular forces arising from hydration (Fe^3+^⋅H_2_O/SO_4_^2−^⋅H_2_O), H-bonds (H_2_O⋅H_2_O) and ion-ion attraction (Fe^3+^⋅SO_4_^2−^). Calculation reveals that Fe^3+^ and SO_4_^2−^ are mostly paired in the absence of ***E*** (supplementary information 8a–d and 7a). As ***E*** is applied the paired Fe^3+^⋅SO_4_^2−^ rapidly move towards electrodes through hydration with H_2_O acting as carriers (blue arrow, supplementary information 8e–h). Pairing also occurs between adjacent S and O (SO_4_^2−^·SO_4_^2−^, supplementary information 9a–c) so excess of anions is dragged into Fe^3+^⋅SO_4_^2−^ clusters and produce unequal number of positive and negative charges at ***P*** and ***N***, *i.e.* the number of cations (Fe^3+^) and anions (SO_4_^2−^) is 22 (= 66 positive charges) and 36 (= 72 negative charges) (red arrow, supplementary information 8 h). In the absence of ***E***, the displacing range of ion-ion clusters is 3–4 nm and decreases to 1–2 nm as ***E*** is applied, attributed to static attraction by electrodes (supplementary information 10a-b). PVA however acts as impermeable objects and prevents cations and anions from pairing, thus facilitating charge separation (complex-II, supplementary information 11a–c and 12a). Calculation indicates the number of cations and anions to be 3 and 2 at ***N*** and 4 and 7 at ***P***, corresponding to 5 positive charges at ***N*** and 2 negative charges at ***P*** (t = 6 s, supplementary information 11d–f and 12b). H-bonds also exist between PVA and anions, accounting for simultaneous movements of both structures toward electrodes (i.e. CH_2_–CHOH⋅OSO_3_^2−^, circle, supplementary information 9c). Owing to large mass, CNTs can barely move and therefore restrict movements of PVA and ion clusters (complex-III, Fig. 7a–b and supplementary information 13a). Aggregation of ions around tubes again verifies physisorption. CNTs however become polarized (white ***N*** and ***P***) as ***E*** is applied and therefore attract more ions, resulting in **I**_**t(tube)**_ > **I**_**t(tube-free)**_ (top and lower, Figs. 4a, 6c,d and supplementary information 13b)^22^.

MD simulation is also carried out on water-free systems that give a large mean free path and reduced elastic scattering of ions, including 2CNTs + 3PVA + Fe^3+^ (**type**-**a**), 2CNTs + 3PVA + SO_4_^2−^ (**type**-**b)** and 2CNTs + 3PVA + Fe^3+^  + SO_4_^2−^ **(type**-**c**) (Fig. 7a–c). Upon ***E*** application, Fe^3+^ rapidly moves toward electrode (white arrow, Fig. 7a) while anion strongly interacts with C–C bonds of CNTs (Fig. 7b). Note that SO_4_^2−^ is a distorted tetrahedral structure where charges locate at O (insert, Fig. 7b). Accordingly, the C–C bonds may electronically correlate with anion and lie possibly between two O-S bonds (**1**) or O–S and O=S bonds (**2**) and two O=S bonds (**3**); the (**2)** however prevails according to MD data (inserts, Fig. 7b). Simultaneous addition of Fe^3+^ and SO_4_^2−^ into CNTs-PVA complexes results in (i) coupling of Fe^3+^ and SO_4_^2−^ and (ii) trapping of Fe^3+^⋅SO_4_^2−^ at intertube groove (Fig. 7c)^23^; the (i) supports Figs. 4 and 6.

## Conclusion

Diode structure is successfully created in composite films made of PVA, Fe_2_(SO_4_)_3(*aq*)_ and CNTs. DL and electrical rectification are verified by elemental analyses and I–V measurements. Calculations confirm ***E*** induced film polarization and charge separation through hydration with H_2_O acting as carriers. Diode character is preserved in strained composite films.

## Supplementary Information

Supplementary Information 1.

Supplementary Video 1.

Supplementary Video 2.

Supplementary Video 3.

Supplementary Video 4.

Supplementary Video 5.

Supplementary Video 6.

## Data Availability

The raw data of Figs. 4 and 5 and simulation videos in this study are available from zenodo.org (10.5281/zenodo.6570681). Other data and material used and analyzed in this study are available from the corresponding author on reasonable request.

## References

[1] Wilson, P. R. Measurements on the depletion layer properties of planar diodes. *Solid·State Electron,* **12**, 539–547. 10.1016/0038-1101(69)90109-9(1969).

[2] Jung YH, Zhang HL, Gong SQ, Ma ZQ (2017). High-performance green semiconductor devices: Materials, designs, and fabrication. Semicond. Sci. Technol..

[3] Roth S, Filzmoser M (1990). Conducting polymers—Thirteen years of polyacetylene doping. Adv. Mater..

[4] Andrews R, Weisenberger MC (2004). Carbon nanotube polymer composites. Curr. Opin. Solid State Mater. Sci..

[5] Lee JS, Oh J, Kim SG, Jang J (2015). Highly sensitive and selective field-effect-transistor NonEnzyme dopamine sensors based on Pt/conducting polymer hybrid nanoparticles. Small.

[6] Lucas AA, Lambin PH, Smalley RE (1993). On the energetics of tubular fullerenes. J. Phys. Chem. Solids.

[7] Schadler LS, Giannaris SC, Ajayan PM (1998). Load transfer in carbon nanotube epoxy composites. Appl. Phys. Lett..

[8] Long RQ, Yang RT (2001). Carbon nanotubes as superior sorbent for dioxin removal. JACS.

[9] Krause B (2011). Influence of dry grinding in a ball mill on the length of multiwalled carbon nanotubes and their dispersion and percolation behaviour in melt mixed polycarbonate composites. Compos. Sci. Technol..

[10] Na PS (2005). Investigation of the humidity effect on the electrical properties of single-walled carbon nanotube transistors. Appl. Phys. Lett..

[11] Peter Atkins, J. d. P. *Physical Chemistry*. P.980 (W. H. Freeman and Company, 2002).

[12] Fujii R, Gotoh Y, Liao MY, Tsuji H, Ishikawa J (2006). Work function measurement of transition metal nitride and carbide thin films. Vacuum.

[13] Lien DH, Hsu WK, Zan HW, Tai NH, Tsai CH (2006). Photocurrent amplification at carbon nanotube-metal contacts. Adv. Mater..

[14] Baughman, R., Zakhidov, A. & Heer, W. Carbon Nanotubes-The Route Toward Applications. *Science (New York, N.Y.)* **297**, 787–792, doi:10.1126/science.1060928 (2002). 10.1126/science.1060928 12161643

[15] Li S (2019). A nanoarchitectured Na_6_Fe_5_(SO_4_)_8_/CNTs cathode for building a low-cost 3.6 V sodium-ion full battery with superior sodium storage. J. Mater. Chem. A.

[16] Watts PCP (2003). Carbon nanotubes as polymer antioxidants. J. Mater. Chem..

[17] Mamontov E (2009). Diffusion dynamics of water molecules in a LiCl solution: A low-temperature crossover. J. Phys. Chem. B.

[18] Byrne MT, Gun'ko YK (2010). Recent advances in research on carbon nanotube-polymer composites. Adv. Mater..

[19] Martí A, Balenzategui JL, Reyna RF (1997). Photon recycling and Shockley’s diode equation. J. Appl. Phys..

[20] McKelvey, J. P. *Solid State and Semiconductor Physics*. (Harper & Row, 1966).

[21] Chan, V. *et al.* in *CICC: Proceedings of the IEEE 2005 Custom Integrated Circuits Conference* 667–674 (2005).

[22] Zhu Y-F (2009). Alignment of multiwalled carbon nanotubes in bulk epoxy composites via electric field. J. Appl. Phys..

[23] Adu CKW, Sumanasekera GU, Pradhan BK, Romero HE, Eklund PC (2001). Carbon nanotubes: A thermoelectric nano-nose. Chem. Phys. Lett..

