# Supplementary material for: Electric field created p–n junction in composite films made from carbon nanotubes, iron (III) sulfate and polyvinyl alcohol
Source: Sci Rep. 2022 Jul 1;12:11203. doi: 10.1038/s41598-022-15294-4 (PMC9249867; doi:10.1038/s41598-022-15294-4)
Supplement: Supplementary file 1 — Supplementary Information 1. [file 41598_2022_15294_MOESM1_ESM.docx]

Supplementary Information

Title Electric field created p-n junction in composite films made from carbon nanotubes, iron (III) sulfate and polyvinyl alcohol

Hsin-Jung Tsai, Ching-You Ke, Hsuan-Hao Huang, Wen-Kuang Hsu^*^

^1^ Department of Materials Science and Engineering, National Tsing-Hua University, Hsinchu City 300044, Taiwan

*Corresponding author e-mail: wkhsu@mx.nthu.edu.tw


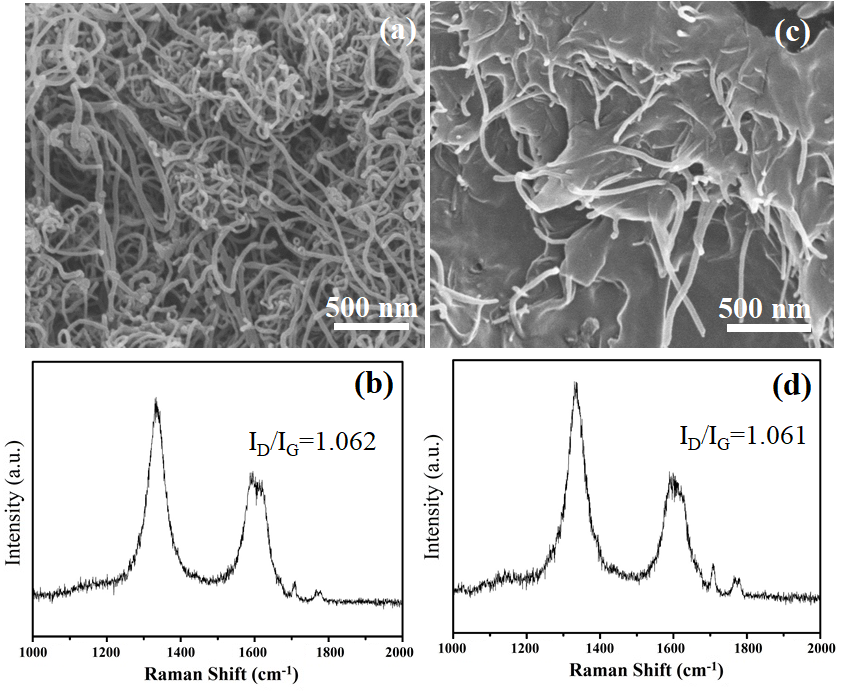


**Fig. S1**. SEM images and raman spectra of multi-walled CNTs before (a, b) and after ball milling treatments (c, d). The I_D_/I_G_ is similar before and after ball milling.

**
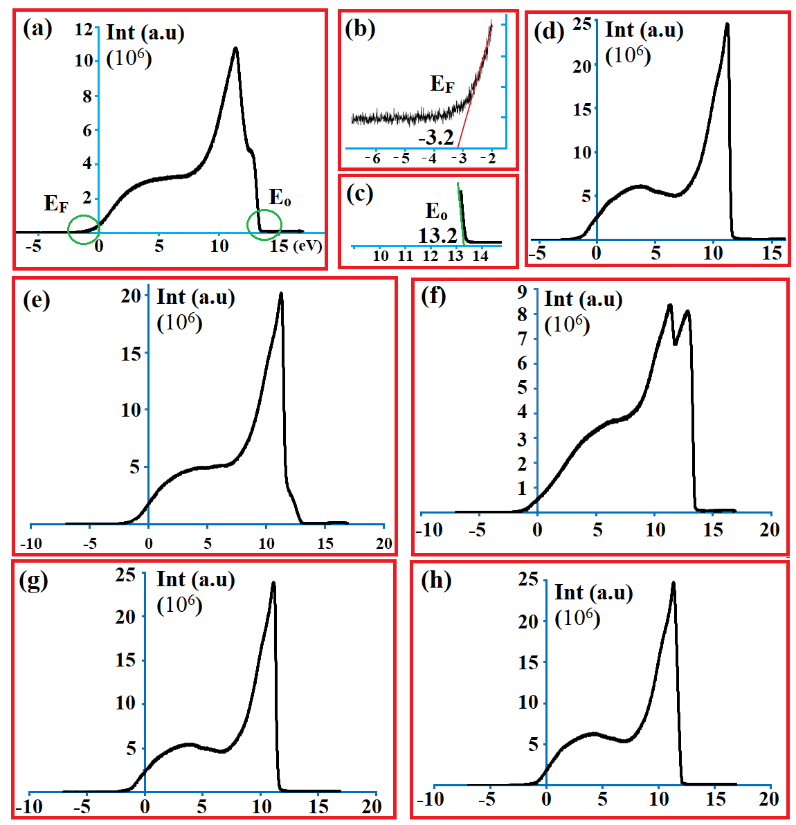
**

**Fig. S2**. UPS spectra of samples made at ***el***-***Conc*** = 0.1 M, *f_CNT_* = 12 w.t% and ***E*** = 0 (a) and determination of E_o_ and E_F_ (b-c). UPS spectra of samples made at ***el***-***Conc*** = 0.1 M, *f_CNT_* = 12 w.t% and ***E*** = 600 V/cm at ***P*** (d) and ***N*** (e). UPS spectra of samples made at ***el***-***Conc*** = 0.5 M, *f_CNT_* = 12 w.t% and ***E*** = 0 (f) and ***E*** = 600 V/cm at ***P*** (g) and ***N*** (h).


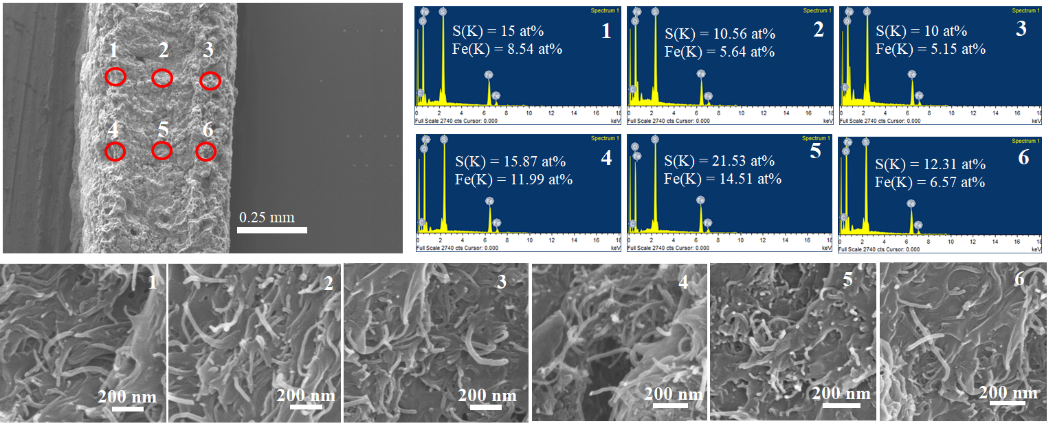


**Fig.S3**. Selected area analyses and SEM images obtained from different regions across film thickness (1-6) where analyses were performed. The energy count is 10^5^ and is taken from K-shell of S and Fe.

**
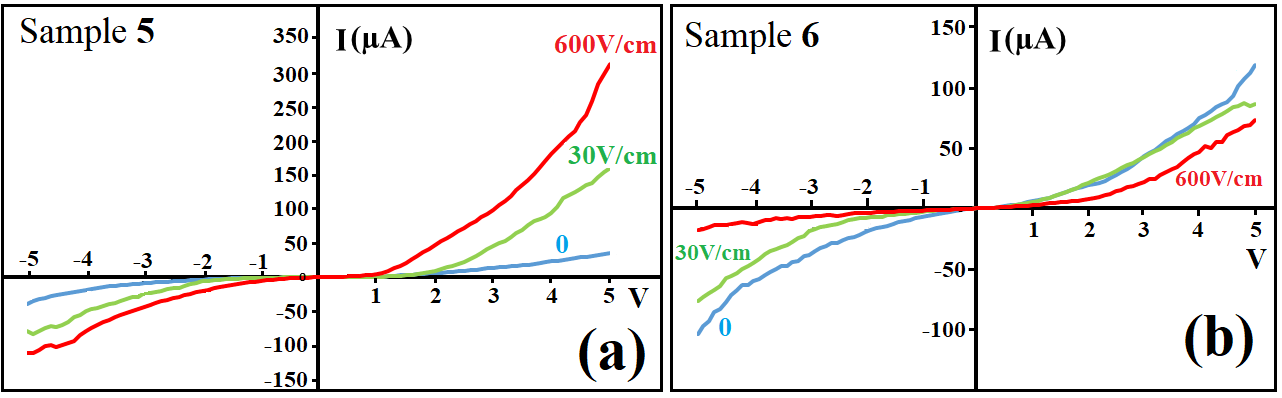
**

**Fig. S4.** The I-V profiles of samples **5** (a) and **6** made at ***E*** = 0, 30 and 600 V/cm (b).


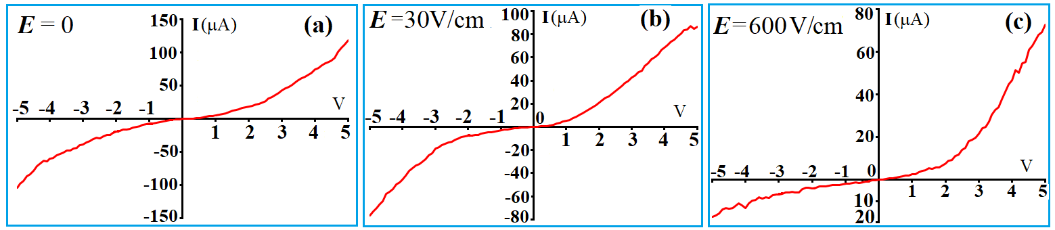


**Fig. S5**. Highlighted I-V profiles of sample **6** made at ***E*** = 0 (a), 30 V/cm (b) and 600

V/cm (c).


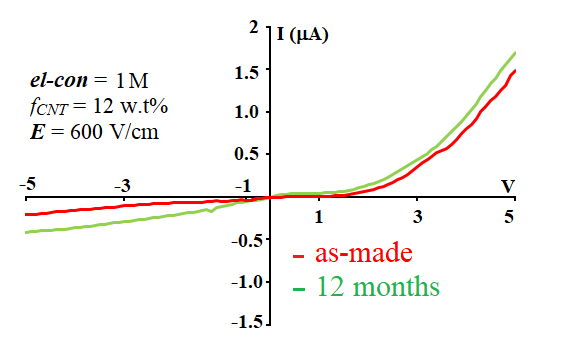


**Fig. S6**. I-V curves of sample made from ***el***-***con*** = 1M, *f_CNT_* = 12 w.t% and ***E*** = 600. Red curve obtained from as-made and over 12 months kept in oven (green).


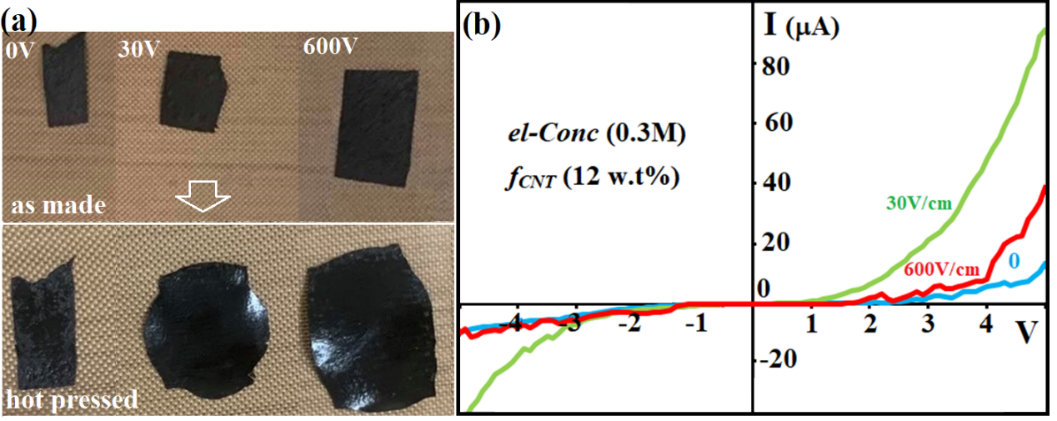


**Fig. S7**. Optical images of samples made from PVA loaded with *f_CNT_* = 12 w.t% and ***el***-***Conc*** = 0.3 M before (top) and after hot-press (lower) (a) and corresponding I-V profiles at ***E*** = 0, 30 and 600 V/cm (b).


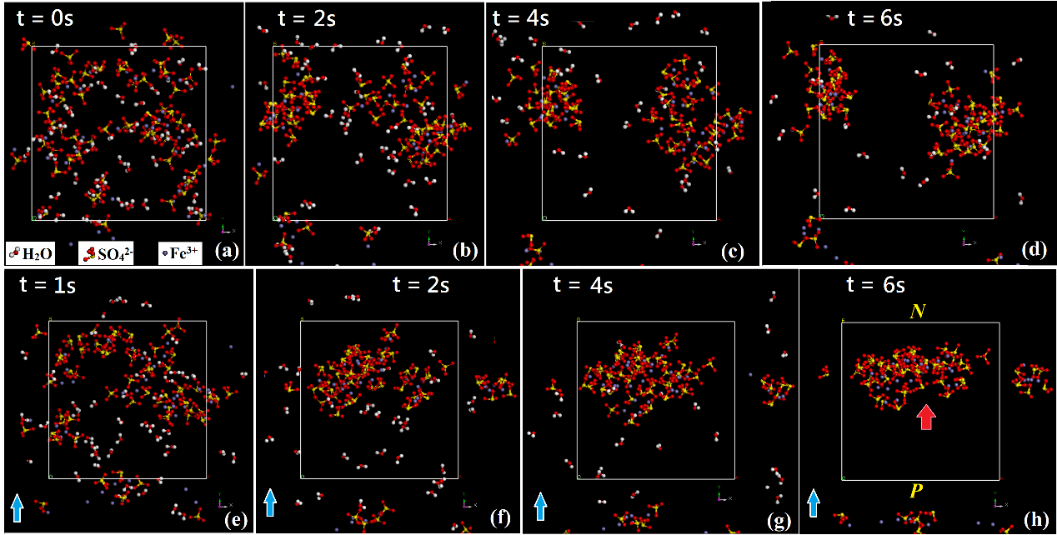


**Fig. S8**. Snapshots extracted from geometric optimization of complexes which consist of 50 H_2_O, 40 Fe^3+^ and 60 SO_4_^2-^ at ***E*** = 0 and t = 0s (a), 2s (b) 4s (c) and 6s (d). Geometric optimization of complexes which consist of 50 H_2_O, 40 Fe^3+^ and 60 SO_4_^2-^ at ***E*** = 600 V/cm and t = 1s (e), 2s (f) 4s (g) and 6s (h).


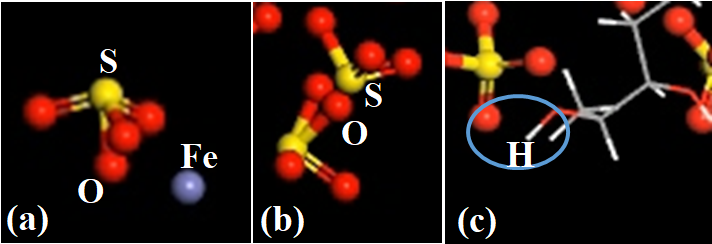


**Fig. S9**. Snapshots extracted from geometrically optimized structures of Fe^3+^···OSO_3_^2-^ (a), S···OSO_3_^2-^ (b) and CH_2_-CHOH···OSO_3_^2-^ (c).

**Supplementary information 10**. Simulation video footages extracted from complexes consisting of 50 H_2_O, 40 Fe^3+^ and 60 SO_4_^2-^ at ***E*** = 0 (a) and 600 V/cm (b).


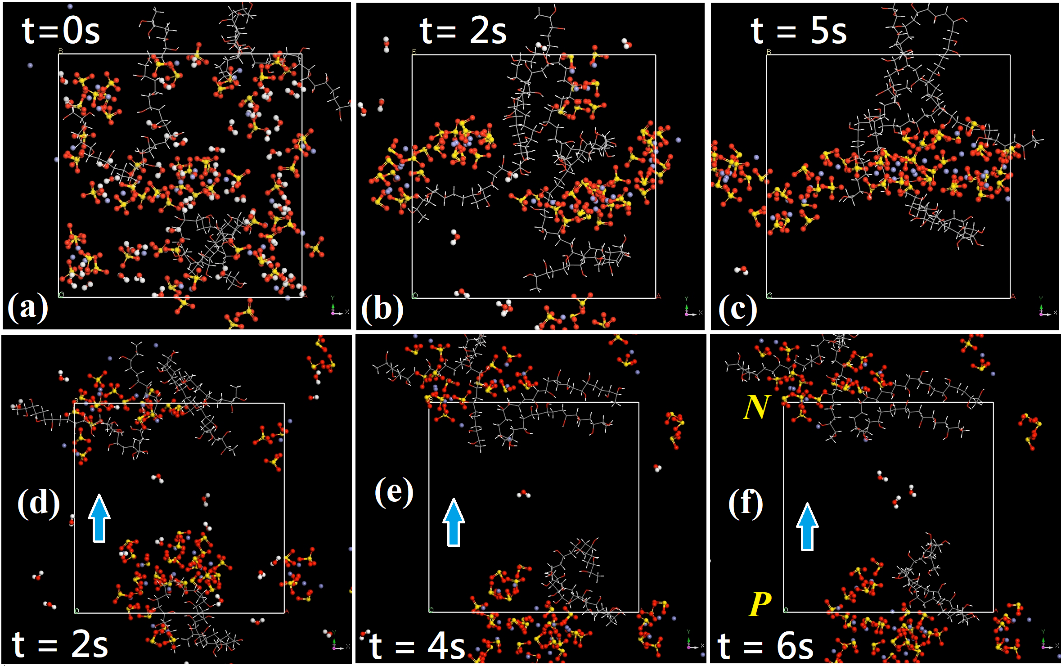


**Fig. S11**. Snapshots extracted from geometric optimization of complexes which consists of 50 H_2_O, 40 Fe^3+^, 60 SO_4_^2-^ and 3PVA at ***E*** = 0 and t = 0s (a), 2s (b) 5s (c) and at ***E*** = 600 V/cm and t = 1s (d), 4s (e) and 6s (f).

**Supplementary information 12**. Simulation video footages extracted from complexes consisting of 50 H_2_O, 40 Fe^3+^, 60 SO_4_^2-^ and 3 PVA at ***E*** = 0 (a) and 600 V/cm (b).

**Supplementary information 13**. Simulation video footages extracted from complexes consisting of 50 H_2_O, 40 Fe^3+^, 60 SO_4_^2-^, 3 PVA and 2 single-walled CNTs (0,8) at ***E*** = 0 (a) and 600 V/cm (b).
